# Combined use of pumpless extracorporeal lung assist system and continuous renal replacement therapy with citrate anticoagulation in polytrauma patients

**DOI:** 10.1186/cc12002

**Published:** 2013-03-19

**Authors:** HK Atalan, M Dumantepe, TB Denizalti, IA Tarhan, A Ozler

**Affiliations:** 1 Atasehir Memorial Hospital, Istanbul, Turkey

## Introduction

The usefulness of a pumpless extracorporeal lung assist system (pECLA) and continuous renal replacement therapy (CRRT) in critically ill patients has been demonstrated in previous studies [1,2]. The aim of this report was to examine combined use of pECLA and CRRT to improve carbon dioxide and inflammatory mediator removal, which allows for lung protective ventilation strategies.

## Methods

**Figure 1:**
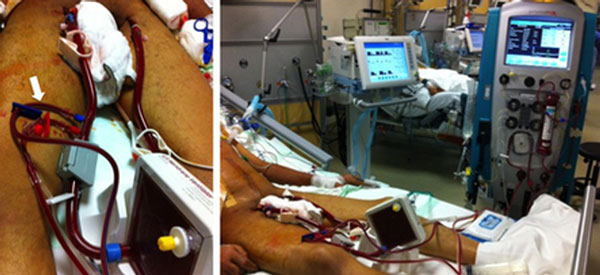
**ILA and CRRT connected to the patient**.

In our 10 patients with ARDS due to polytrauma and sepsis, pECLA was established by insertion of cannulae to the femoral artery and vein. CRRT cannulae were introduced by venous line of the same vascular access (Figure 1). We preferred regional anticoagulation with trisodium citrate for both CRRT and ILA.

## Results

**Figure 2:**
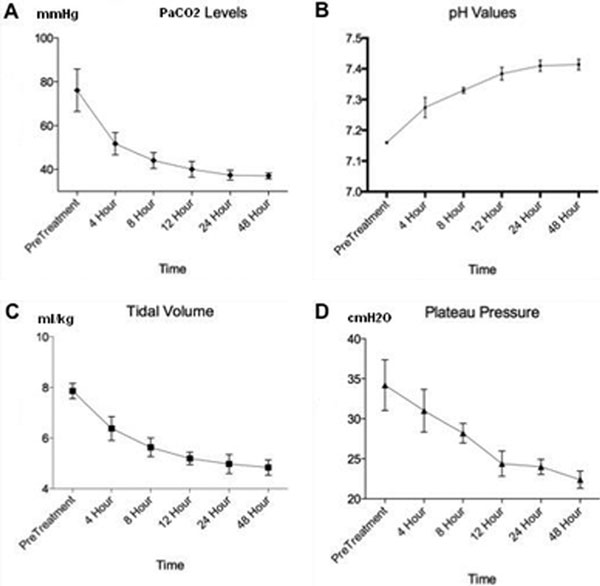
**Changes in tidal volume, plateau pressure, pH and PaCO_2_**.

Mean SAPS II and APACHE II scores were 55 and 23 respectively. Mean time on mechanical ventilation was 22 days. Mean ICU stay was 30 days for survivors and 38 days for nonsurvivors. When compared with baseline values most relevant parameters were the improvement in tidal volumes, plateau pressures, PaCO_2 _levels and pH (Figure 2). Four patients survived while six patients died from sepsis-MOF.

## Conclusion

We concluded that pECLA can effectively address the impaired gas exchange in ARDS and CRRT is a safe procedure with potential therapeutic value for treating MOF. Citrate anticoagulation was well tolerated and filter life was appropriate. The use of the same vascular access for ILA and CRRT may minimize invasive procedures and related side effects.
